# Sulconazole inhibits PD-1 expression in immune cells and cancer cells malignant phenotype through NF-κB and calcium activity repression

**DOI:** 10.3389/fimmu.2023.1278630

**Published:** 2024-01-05

**Authors:** Simon Pernot, Mercedes Tomé, Isabel Galeano-Otero, Serge Evrard, Iker Badiola, Frederic Delom, Delphine Fessart, Tarik Smani, Geraldine Siegfried, Bruno O. Villoutreix, Abdel-Majid Khatib

**Affiliations:** ^1^ Reprograming tumor activitY and associaTed MicroenvironmEnt (Rytme), Bordeaux Institute of Oncology (BRIC)-UMR1312 Inserm, Université of Bordeaux, Pessac, France; ^2^ Institut Bergonié, Bordeaux, France; ^3^ Department of Cell Biology and Histology, Faculty of Medicine and Nursing, University of the Basque Country (UPV/EHU), Leioa, Spain; ^4^ Group of Cardiovascular Pathophysiology, Institute of Biomedicine of Seville, University Hospital of Virgen del Rocío/University of Seville/CSIC, Seville, Spain; ^5^ Integrative Computational Pharmacology and Data Mining, INSERM UMR 1141, Rob-ert-Debré Hospital, Paris, France

**Keywords:** PD-1, Jurkat T cells, PBMCs, NF-κB, calcium, cancer, zebrafish

## Abstract

The overexpression of the immunoinhibitory receptor programmed death-1 (PD1) on T-cells is involved in immune evasion in cancer. The use of anti-PD-1/PDL-1 strategy has deeply changed the therapies of cancers and patient survival. However, their efficacy diverges greatly along with tumor type and patient populations. Thereby, novel treatments are needed to interfere with the anti-tumoral immune responses and propose an adjunct therapy. In the current study, we found that the antifungal drug Sulconazole (SCZ) inhibits PD-1 expression on activated PBMCs and T cells at the RNA and protein levels. SCZ repressed NF-κB and calcium signaling, both, involved in the induction of PD-1. Further analysis revealed cancer cells treatment with SCZ inhibited their proliferation, and migration and ability to mediate tumor growth in zebrafish embryos. SCZ found also to inhibit calcium mobilization in cancer cells. These results suggest the SCZ therapeutic potential used alone or as adjunct strategy to prevent T-cell exhaustion and promotes cancer cell malignant phenotype repression in order to improve tumor eradication.

## 1 Introduction

The inhibitory receptor programmed cell death protein-1 (PD-1, PDCD1) is a member of the immunoglobulin (lg) superfamily (1). The expression of this type I transmembrane protein is induced in various immune cells including T cells, B cells, macrophages, and several dendritic cell subsets (2–4). Two main ligands of PD-1, namely programmed cell death ligand 1 and 2 (PD-L1/PD-L2) (5) are expressed in various tumor cell types and immune cells (5, 6). By binding to PD-1, PD-L1 inhibits T cell activation, leading to immune escape. Thereby, interfering with PD-1 and PD-L1 interaction or signaling pathways was proposed as a therapeutic approach to prevent cancer cells from avoiding the anti-tumoral immune response. This by reactivating the T-cell-mediated tumor cell cytotoxicity and elimination (1–5). Indeed, previous studies indicated that the inhibition of PD-1 promotes an effective immune response against cancer cells (1–5) and targeting PD-L1 or PD-1 using monoclonal antibody blocking PD-1 or PD-L1 has been associated with significant clinical response in a wide range of malignancies (7, 8). However, although blockade of PD-1/PD-L1 with these monoclonal antibodies shows therapeutic effect for cancer patients, their use displayed some restrictions. These include the reduced response frequency and several adverse effects observed in some cancer patients (7, 8). Therefore, to overcome these difficulties the development of other efficient strategies is now necessary.

Antifungal imidazole derivatives are commonly used for the treatment of topical and systemic infections including candidal infections and mycoses (9). Imidazole derivatives, including ketoconazole (KET), miconazole (MIC), tioconazole (TIO), clotrimazole (CLO), and sulconazole (SCZ), were initially identified as ligands of the heme iron atom of cytochrome P450 (CYP) (10–12). Furthermore, the effect of these drugs have been studied in the context of human pathologies treatment including cancer (13). Of these, SCZ is an antifungal agent with a broad spectrum of activity and is proposed for the treatment of skin infections such as dermatophyte infections (14–16). Compared to several imidazoles, SCZ shows enhanced antifungal activity (14–16) and was reported to inhibit the malignant phenotype of breast cancer cells (13). In this study we demonstrate the ability of SCZ to inhibit the expression of PD-1 on activated T cells and PBMCs through NF-κB activity and calcium mobilization repression. Sulconazole was also able to inhibit colon cancer and breast cancer cells as well as melanoma proliferation, and migration, suggesting the dual therapeutic effect of this drug.

## 2 Materials and methods

### 2.1 PBMCs, T cells and cancer cells culture and PBMCs activation

Human peripheral blood mononuclear cells (hPBMCs) were obtained following written informed consent approved by Bergonié Institute (Bordeaux, France). PBMCs were isolated from healthy donors by density gradient centrifugation with Pancoll (PANBiotech; human, density 1,077g/ml) and were cultured in RPMI 1640 (PAN-Biotech) supplemented with 10% FBS (Gibco), 2mM L-glutamine (Gibco) and penicillin/streptomycin solution (Dominique Dutscher). Human T cells were purified from blood using the MACSxpress® Whole Blood Pan T Cell Isolation Kit (Miltenyi Biotec). The murine colon cancer cells CT-26, the human colon cancer cells HT29, melanoma cells M10 and Jurkat T and J.RT3-T3.5 (JRT3) cell lines were cultured in RPMI 1640 complete media. The breast cancer cells MDA-MB 231 were cultured in DMEM complete media. All the cells were grown at 37°C in a 95% air, 5% CO2 humidified incubator. Activation of Jurkat T cells, purified T cells and PBMCs was induced by phorbol myristate acetate (PMA,100ng/ml) and Ionomycin (Io,1ug/ml), as previously described (17).

### 2.2 Real-time qPCR

Total RNA was isolated using the Nucleospin RNA kit (Macherey-Nagel) prior reverse transcription in a reaction mixture containing 50mM Tris-HCl (pH 8.3), 30mMKCl, 8mM MgCl2, 1mM dNTPs, and 0.2U Superscript reverse transcriptase (Invitrogen), as previously described (17, 18) in a VeritiThermal Cycler (Applied Biosystem). Quantitative real-time PCR was performed using specific TaqMan primers and Master Mix (Eurogentec), in a StepOne Plus Real-Time PCR system following manufacturer’s instructions (Applied Biosystem). GAPDH was used for normalization. The PDCD1 primers used are F 5’-CTACAACTGGGCTGGCGG-3’ and R 5’-TGTGTTGGAGAAGCTGCAGG-3, respectively. The primers for CTLA4 were derived from BioRad Unique Assay (ID qHsaCED0003794).

### 2.3 Ca^2+^ mobilization measurement quenching assay

Indicated cells were treated for 48 h with 10 µM of Sulconazole or vehicle (DMSO) in their culture media, and then Ca^2+^ influx was evaluated using 2 µM Fura-2 AM (ThermoFisher Scientific, US) and microfluorometry system, for adherent cells, or CLARIOstar^®^ Plus microplate reader (BMG Labtech; Germany), for T cells. The microfluorometry system includes inverted microscope Leica (Wetzlar, Germany) with a 20×/0.75 NA objective, a monochromator (Polychrome V, Till Photonics, Munich, Germany), a CCD camera and HP software (Hamamatsu Photonics, Japan). In both cases, the changes of intracellular Ca^2+^ concentration ([Ca^2+^]_i_) were shown as the ratio of Fura-2 AM fluorescence after excitation at 340 and 380 nm (ratio = *F_340_/F_380_*). Experiments were done following the period sequence: 4 min in (1) free Ca^2+^ solution (140 mM NaCl, 2.7 mM KCl, 4 mM MgCl_2_, 0.5 mM EGTA, 10 mM HEPES, pH = 7.4) with 2 µM of thapsigargin, in order to stimulate Store Operated Ca^2+^ Entry (SOCE), and 6 min in (2) 2-2.5 mM Ca^2+^ solution (2-2.5 mM CaCl_2_; 140 NaCl, 2.7 KCl, 1 MgCl_2_, 0.5 EGTA, 10 HEPES, pH = 7.4). Then, Ca^2+^ influx (Δratio) was computed as the difference between the peak ratio after 2 min of extracellular Ca^2+^ re-addition and its level just before.

### 2.4 JRT3 functional assay

This functional assay, is based on Jurkat T-cell line stably expressing the human LES -γδ TCR (JRT3-LES) incubated with the colon cancer cell line HT29 overexpressing the endothelial protein C receptor (HT29-EPCR), as previously described (17). The activation of JRT3-LES cells was evaluated by the expression of CD69.

### 2.5 Proliferation assay

The proliferation of indicated cells was performed using the IncuCyte live-cell microscopy incubator (Essen Bioscience). Cells (2 × 10^5^) were treated with indicated concentrations of SCZ in the presence of 3% and 10% serum and placed in the IncuCyte incubator, and phase-contrast images were taken at regular intervals over 96 hours. Results were calculated by the IncuCyte software and presented as confluence relative to time 0. Images were taken with a ×4 objective. Four images were taken from each well, and each condition had more than 6 wells.

### 2.6 Wound healing assay

Cells were seeded in 96-well plates and allowed to grow until they reached 90% confluence (2 × 10^6^ cells per well of each cell line). The cell wound was performed using Incucyte® Wound Maker 96-Tool, and cells were treated with various concentration of SCZ during various time periods. Plates were placed in the IncuCyte incubator which took 4 phase-contrast images and calculated cell-free area of each well at regular intervals over 48 hours. Results were shown as relative wound density.

### 2.7 Immunocytochemistry

Detection of PD-1 in Jurkat T cells were monitored using an anti-PD-1 antibody at 1:100 in TBS with 5% BSA, as previously described (17). Confocal immunofluorescence images were taken using the inverted microscope Nikon C2si Eclipse Ti-S with NIS-ElementsAR software (NikonInstruments Europe B.V.).

### 2.8 Flow cytometry analysis

Cells were stained with fluorophore-conjugated antibodies: PE-anti-PD-1 mAb (MIH4, #560908 eBiosciences) and Flowcytometry data were acquired with BD Accuri C6 (BD Biosciences). Flow cytometry analyses were performed using BD Accuri C6 software and FlowJo 9.3.2 (TreeStar), as previously described (17).

### 2.9 Western blotting

Cells were lysed in PBS containing 2% NP-40 and lysates were subjected to SDS-PAGE (BioRad Miniprotein) and proteins were blotted onto polyvinylidene difluoride membrane (PVDF, Amersham Pharmacia Biotech). The primary antibodies anti-NF-κB p65 (#sc-8008, dilution 1/500) from Santa Cruz Biotech, phospho-NF-κBp65 (#3033, dilution 1/500) from Cell signaling, TRPC1: rabbit anti-TRPC1 (1:500, T8276; Sigma-Aldrich, United States), STIM1: rabbit anti-STIM1 (1:500, 4916S; Cell Signaling, United States) and Orai1: rabbit anti-Orai1(1:250, O8264; Sigma-Aldrich, United States) were revealed by HPR-conjugated secondary antibodies (Amersham Pharmacia Biotech) and enhanced chemiluminescence (Pierce ECL Plus, Thermo Scientific) according to the manufacturer’s instructions. Images were acquired with a Genegnome system and GeneSyssoftware (Syngene) (17–19).

### 2.10 Tumorigenicity assay

All experiments performed in this study were approved by the university of Bordeaux Animal Ethics. Adult AB zebrafish strain (ZIRC, USA) were maintained following the French Directive under permission number A33-063-935. All the procedures were conducted in compliance with the European Communities Council Directive (2010/63/EU). Following the production of embryos by adult zebrafish, embryos were allowed to grow in E3 medium (5 mM NaCl, 0.17 mM KCl, 0.33 mM CaCl2, 0.33 mM MgSO4) at 28°C, as described previously (20, 21). After dechorionation, 2-day post fertilization (dpf) zebrafish embryos were anaesthetized with 0.003% tricaine (Sigma, USA) and placed in 3% methylcellulose on a dish coated with 1% agarose. MDA-MDB-231 cells were detached using Versaine solution and 2.5·10^6^ cells were resuspended in 50 μl of PBS containing 1% phenol red. Immediately, cell suspension was loaded into Femtotip II capilar needles (Eppendorf, Ge**r**many) and injections were performed using a pump (Femtojet 4i; Eppendorf, Germany) and a micromanipulator (Phymep). Around 500 cells per embryos were inserted above the duct of Cuvier in perivitelline space of the embryo, as previously described (22). After checking the implantation with mammalian cells, zebrafish embryos were maintained in 24-well plates at 36.3°C. Then, SCZ (1 µM) or vehicle (DMSO) we added of to each well. Tumor imaging was done after 48-h post injection (hpi) using Nikon EclipseTS100 microscope. The tumor size was evaluated using the area of the developed tumors.

### 2.11 Statistical analysis

Unless otherwise indicated, data are presented as mean ± SEM. A 2-tailed t test was used to analyze the data in GraphPad Prism (GraphPad Software). The statistical significance level is illustrated with P (p-values). Statistical P was set than 0.05.

## 3 Results

### 3.1 Sulconazole inhibits PD-1 expression in T cells and PBMCs

**Figure 1:**
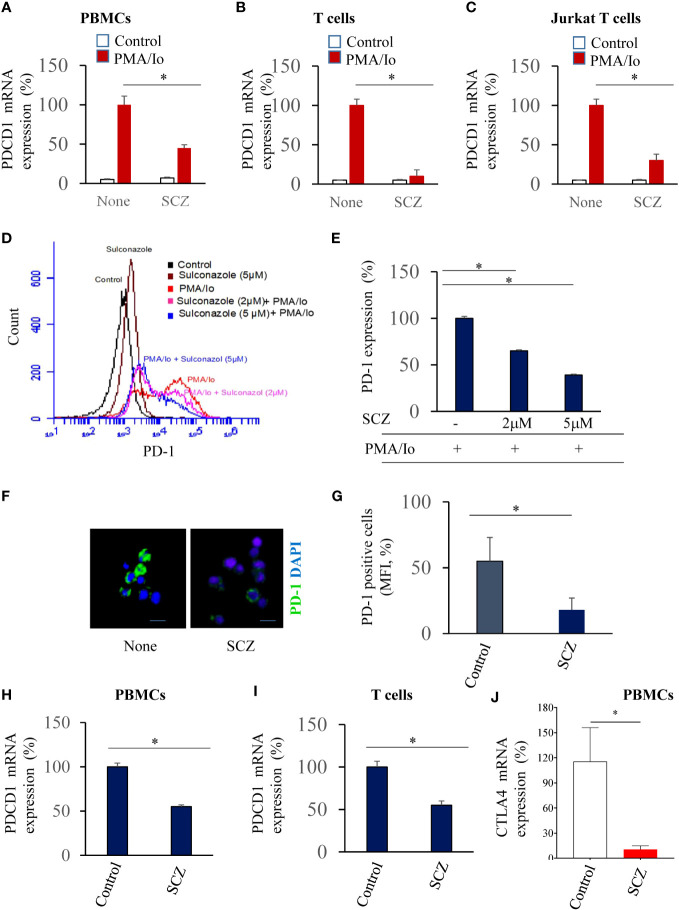
Inhibition of PDCD1 expression in PBMCs **(A)**, purified T cells **(B)** and Jurkat T cells **(C)** by Sulconazole (SCZ). PD-1 mRNA level upon PMA/Io stimulation in the absence and presence of SCZ (5μM) at 24h, assessed by real time-PCR. Data are represented as fold change to PMA/Io-stimulated cells that was assigned 100% as mean ± SEM from three independent experiments. *, *P* < 0.05. **(D, E)** Jurkat T cells were treated with indicated SCZ concentrations prior their incubation with PMA/Io. PD-1 expression was analyzed by Flow cytometry. **(F, G)** Representative confocal microscopy images of PD-1 immunostaining (green) of PMA/Io-activated Jurkat T cells in the absence and presence of SCZ. **(H, I)** PD-1 expression in PBMCs **(H)** and purified T **(I)** cells treated with SCZ prior their incubation with PMA/Io, as assesses by Flow cytometry. **(J)** CTLA-4 mRNA level upon PMA/Io stimulation in the absence and presence of SCZ (5μM) at 24h, assessed by real time-PCR. Scale bar, 10 μm. Data are represented as mean ± SEM from three independent experiments. MFI, mean fluorescence intensity (arbitrary unit). * P < 0.05.

A potent T cell response requires PKC signaling (23). To induce PBMCs, human purified T cells activation we first used combination of PMA and Io (PMA/Io) that mimics T-cell receptor (TCR) activation. Indeed, PMA binds to and activates PKC whereas ionomycin (Io) is a calcium ionophore that enhances membrane permeability to calcium. Incubation of indicated cells with PMA/Io for 24h induced PD-1 mRNA expression in all indicated cells (**Figures 1A, B**). In the presence of SCZ (5μM), PD-1 mRNA expression was considerably decreased after 24h of treatment, as assessed by real time-PCR. Similarly, using the Jurkat T cells, their treatment with SCZ, also affect PD-1 expression (**Figure 1C**). These observations indicate that PD-1 expression on T cells and PBMCs can be repressed.

To evaluate the expression of PD-1 in PBMCs and T cells at the protein level, we first treated the PMA/Io activated-Jurkat T cells with indicated SCZ concentrations. Flow cytometry analysis revealed that PD-1 expression is also induced at the protein level in the presence of PMA/Io that was repressed by sulconazole in a dose-dependent manner (**Figures 1D, E**). PD-1 expression was reduced by up to 60% with 5μM of SCZ. The use of immunofluorescence staining under these conditions confirmed that PD-1 expression was greatly decreased in the presence of sulconazole (**Figures 1F, G**). The use of PMA/Io-treated-PBMCs and purified T cells also revealed their reduced PD-1 expression in the presence of SCZ (**Figures 1H, I**). These observations indicate that SCZ can impede PD-1 expression at the RNA and protein levels in PBMCs and T cells. To evaluate whether SCZ is also able to affect the expression of other immune checkpoint inhibitors involved in T cell exhaustion, such as CTLA-4. As illustrated in **Figure 1J**, treatment of activated PBMCs with SCZ (5μM), significantly affected CTLA-4 expression.

### 3.2 Repression of NF-κB expression and calcium mobilization by sulconazole

**Figure 2:**
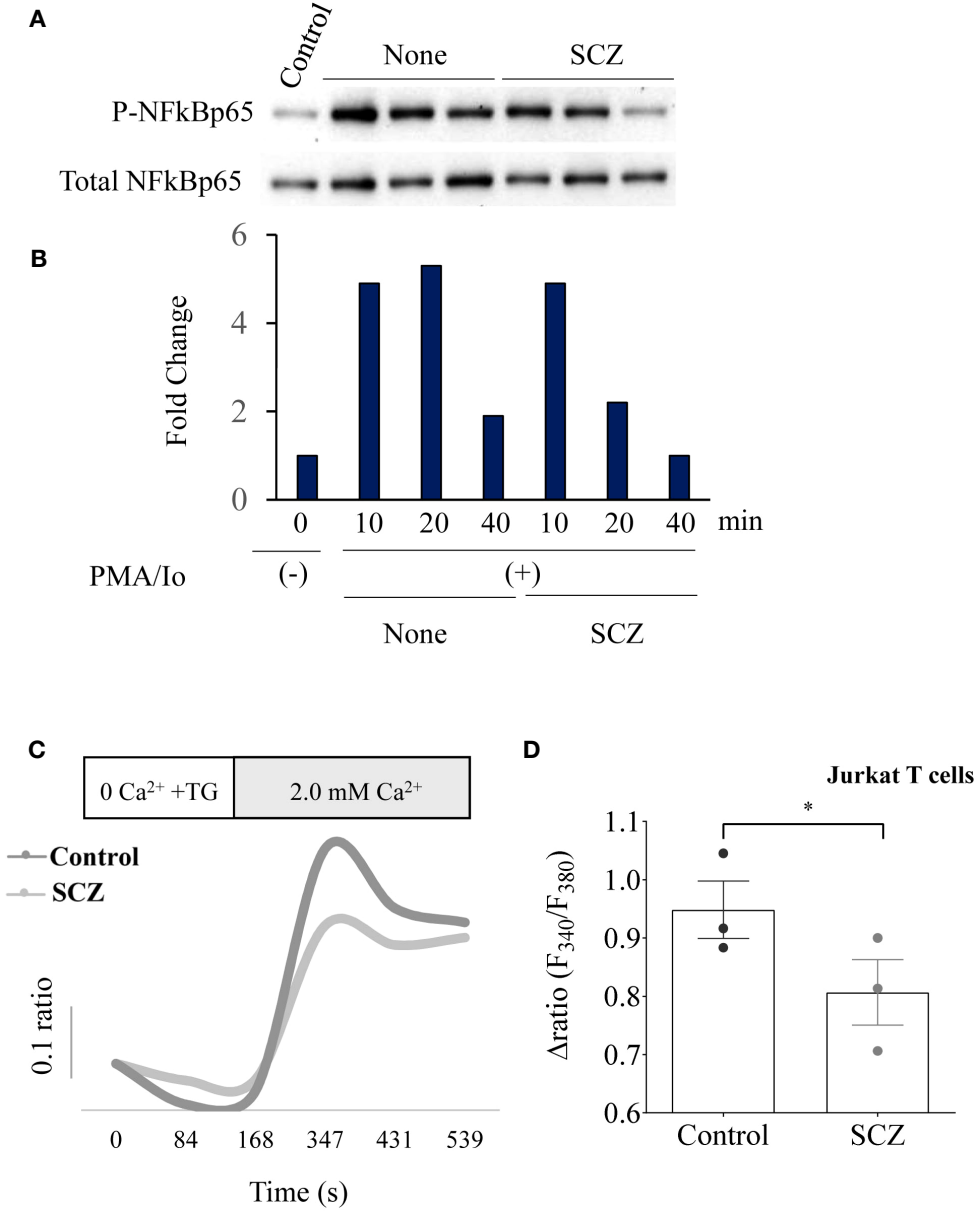
Inhibition of NF-κB phosphorylation and calcium mobilisation by Sulconazole (SCZ) in Jurkat T cells. **(A)** Jurkat T cells were treated with SCZ prior their incubation with PMA/Io and the phosphorylation of NF-κB was analyzed by western blotting at the indicated time points. **(B)** Results of NF-κB phosphorylation quantification are shown in the bar graph and calculated, as the ratio of p-NF-κB/Total NF-κB and are representative of three independent experiments. **(C)** Representative recordings of thapsigargin-induced changes in the intracellular calcium concentration expressed as fluorescence ratio (F340/F380). Jurkat cells were incubated for 4 min in a free Ca^2+^ solution in the presence of 2μM of thapsigargin and Ca^2+^ (2.0 mM) was re-added. **(D)** Bar graph shows the percentage of delta ratio increase after and before adding Ca^2+^ in cells treated with or without SCZ (10μM). Data are represented as mean ± SEM from three independent experiments. * P < 0.05.

NF-kB activation and calcium mobilization has previously been reported to be involved in PD-1 expression (17, 24). Thereby, we next investigated the effect of SCZ on these two PD-1 signaling pathways. As illustrated in **Figures 2A, B** incubation of Jurkat T cells at the indicated time points with PMA/Io induced NF-κB phosphorylation. Maximal stimulation was observed after 10-20 min and was downregulated after 40 min of cells stimulation. The presence of SCZ significantly reduced NF-κB activation. The inhibitory effect of SCZ was significant after 20 min and maximal after 40 min of cells incubation. These results show that SCZ represses the activation of NF-κB required for PD-1 expression. Similarly, store-operated Ca^2+^ entry (SOCE) is a mechanism for Ca^2+^ influx across the plasma membrane activated in response to depletion of intracellular Ca^2+^ stores, mostly in the ER. To evaluate the effect of SCZ on Ca^2+^ mobilization, Jurkat T cells were incubated with sulconazole (10 μM), and Ca^2+^ levels were measured. Cells were loaded with fura-2, and then stimulated with 2 μM thapsigargin to deplete the ER and activate SOCE. As illustrated in **Figures 2C, D**, SCZ inhibited Ca^2+^ entry into cells. These observations, together with the finding that SCZ was able to regulate NF-κB activation, indicate SCZ ability in the repression of different pathways involved in the PD-1 expression on T cells.

### 3.3 Functional and survival of Jurkat T cells in the presence of sulconazole

**Figure 3:**
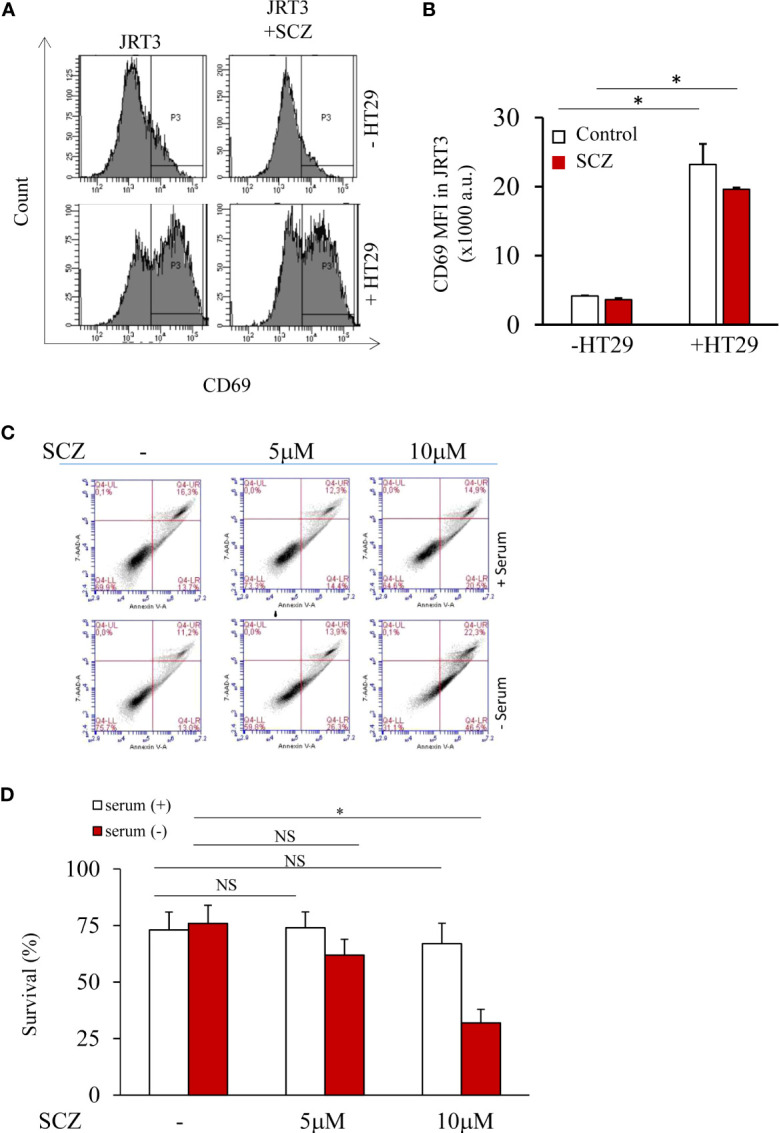
T cell activity and survival are not affected by sulconazole. **(A, B)** Flow cytometry analysis of TCR-activation marker CD69 in JRT3 cells upon binding to HT29 cancer cells in the presence and absence of SCZ **(C, D)** Fluorescence-activated cell sorter scatter plots of Jurkat T cells incubated for 24h without or with serum (5%) at indicated SCZ concentrations. After incubation, cells were double stained with annexin V and 7AAD. The use of fluorescence-activated cell sorter detected viable (negative for both dyes; lower left), early apoptotic (Annexin+/7AAD−, lower right), necrotic cells (Annexin−/7AAD+, upper left) and late apoptotic (Annexin+/7AAD+, upper right) cells. Data represented as mean ± SEM from three independent experiments. *, *P* < 0.05. NS, not significant.

To evaluate the effect SCZ on Jurkat T cells function, we next analyzed the expression of CD69, a T-cell activation marker, by flow cytometry to analyze antigen-TCR binding and therefore, downstream T-cell activation. Thereby we used the TCR-deficient Jurkat T cells (JRT3), expressing a specific TCR (LES) that recognizes the EPCR protein that were cocultured with EPCR-expressing HT29 cancer cells. As illustrated in **Figures 3A, B** the expression of CD69 was upregulated when JRT3-LES cells were cocultured with EPCR-expressing HT29 cells. Similarly, Following JRT3-LES treatment with SCZ (5μM) for 24 hours in the presence of EPCR-expressing HT29 cancer cells also strongly upregulated CD69 expression (**Figures 3A, B**). We next evaluated the effect of SCZ on cell survival. Thereby, T cells were incubated with SCZ in the absence and presence of serum and flow-cytometric analysis of cell death was performed using annexin V and 7AAD as markers. Flow cytometric analysis revealed that Jurkat T cells incubation with up to 5 μM SCZ had no effect on cell survival. In the absence of serum, only the concentration of 10μM induced cell death (**Figures 3C, D**).

### 3.4 Sulconazole inhibits calcium mobilization in cancer cells

**Figure 4:**
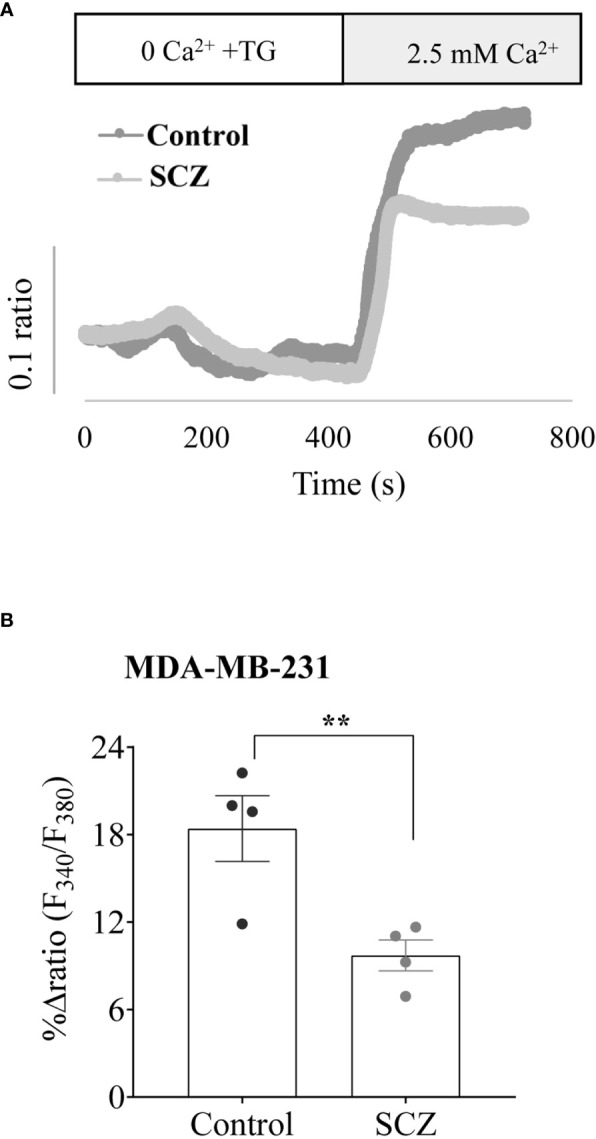
Inhibition of calcium mobilisation by Sulconazole in cancer cells. **(A)** Representative recordings of thapsigargin-induced changes in the intracellular calcium concentration expressed as fluorescence ratio (F340/F380). MDA-MB-231 cells were incubated for 4 min in a free Ca^2+^ solution in the presence of 2μM of thapsigargin and Ca^2+^ (2.5 mM) was re-added. **(B)** Bar graph shows the percentage of delta ratio increase after and before adding Ca^2+^ in cells treated with or without SCZ (10μM). Data are represented as mean ± SEM from four independent experiments.

Calcium is considered as a regulator of the malignant phenotype of cancer cells, therefore, we further investigated the effects of SCZ on Ca^2+^ influx. First, the breast cancer cells MDA-MB-231 were pretreated with SCZ and then calcium signals were detected. As shown in **Figures 4A, B**, Ca^2+^ influx induced by thapsigargin was blocked by SCZ (10 μM). These results implied that SCZ blocked calcium mobilization, a signal pathway required for various processes required for the acquisition of the malignant phenotype of cancer cells, including cell proliferation, and migration. Further analysis revealed that the effect of SCZ on calcium mobilization is not mediated by changes in the levels of the calcium channels STIM1, ORAI1 and TRPC1, as assessed by western blotting analysis (**Supplementary Figure 1**).

### 3.5 Sulconazole inhibits cancer cell proliferation and migration

**Figure 5:**
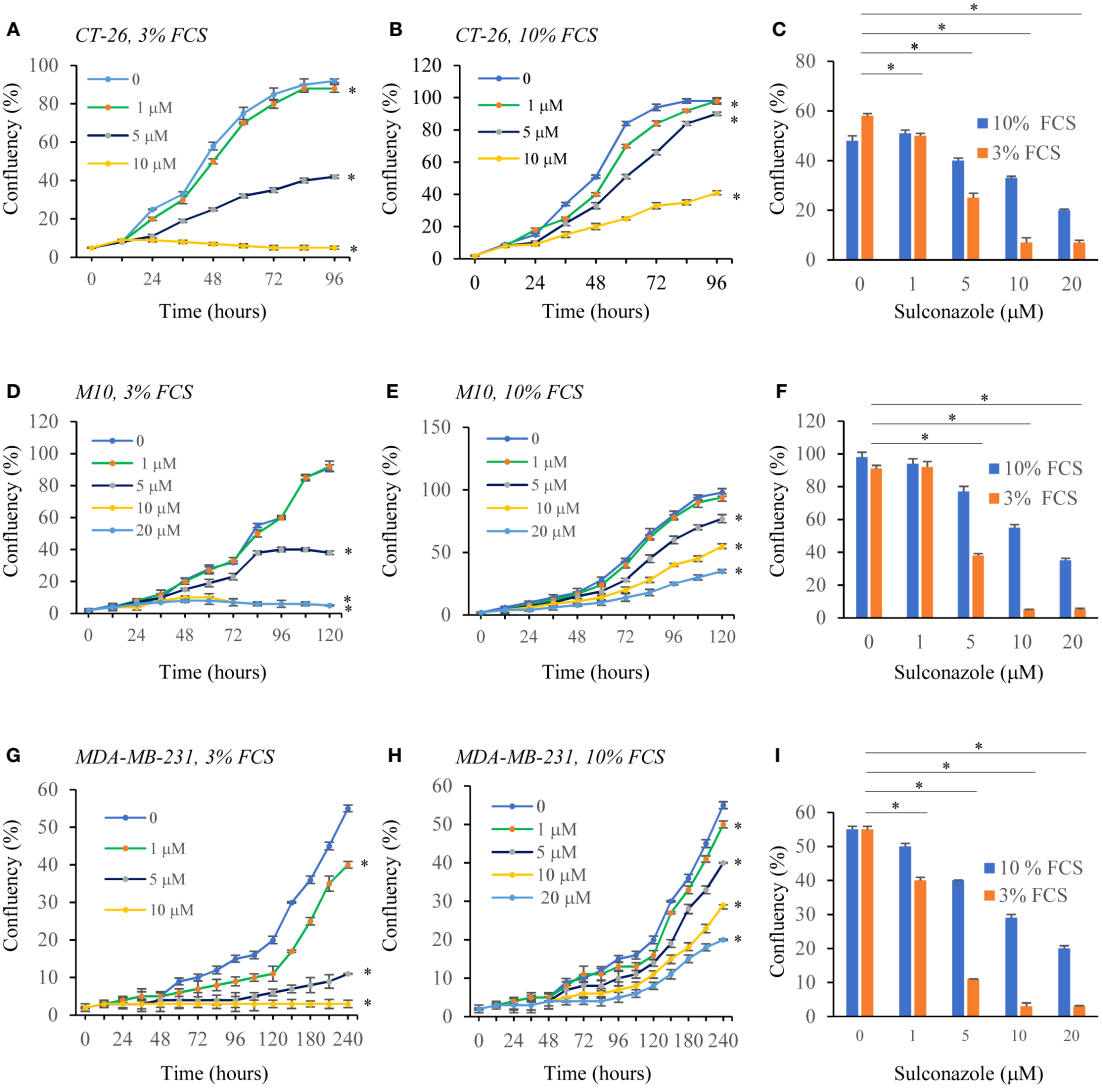
Inhibition of cancer cells proliferation by Sulconazole. colon cancer cells (CT-26) **(A–C)**, Melanoma cells M10 **(D–F)** and breast cancer cells MDA-MB-231 **(G–I)** and were plated at low confluence for time-lapse phase-contrast videomicroscopy (IncuCyte microscope) in the absence and presence of different concentrations of SCZ and serum, and cell proliferation was monitored by automated confluence analysis at set intervals after plating. Data are represented as mean ± SEM from three independent experiments (*n* = 6 wells per group). * P < 0.05.

**Figure 6:**
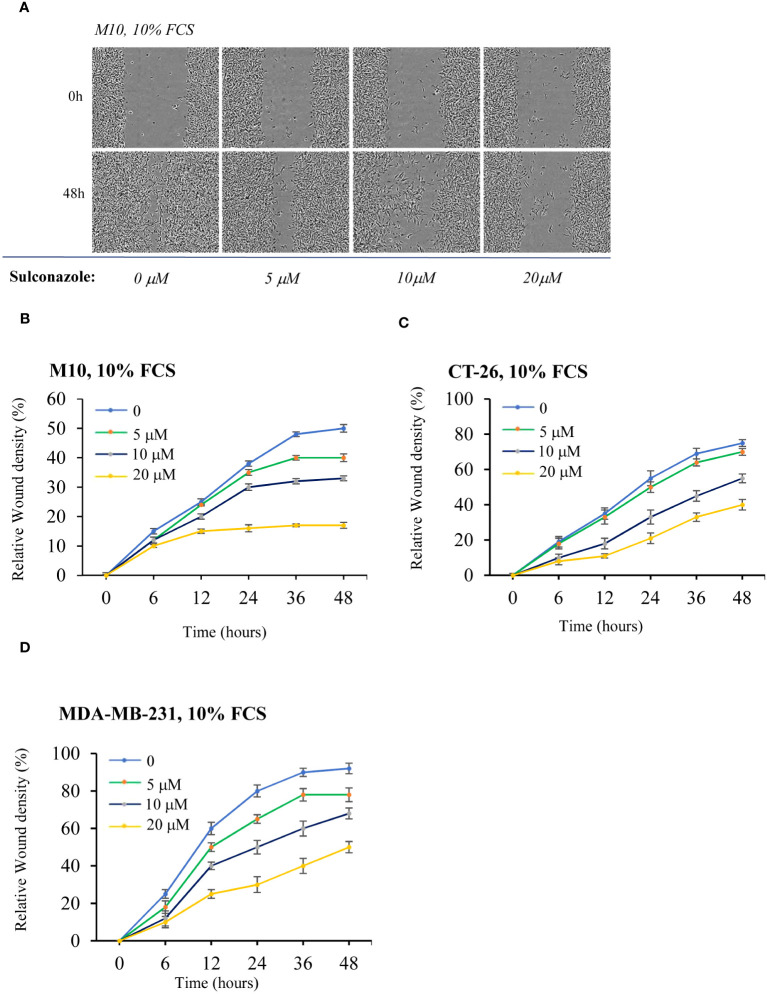
Inhibition of cancer cells migration by Sulconazole. **(A–D)** cancer cell migration was analyzed by scratch wound assay. M10 **(A, B)** CT-26) **(C)**, and MDA-MB-231 **(D)** cells were treated with various SCZ concentrations and were subjected to scratch wounds and imaged using IncuCyte microscope during 48h (*n* = 6 wells per group, 3 independent experiments). **(B–D)** Quantification of wound closure during indicated time periods. Data are represented as mean ± SEM.

To determine the effect of SCZ on the malignant phenotype, the proliferation of several cancer cells, including the melanoma M10, the breast MD-MB231 and the colon CT-26 cancer cell lines were treated with indicated concentrations of SCZ in the presence of low (3%) and high (10%) concentration of serum and their proliferation was measured using the IncuCyte Live Cell Analysis System (**Figures 5A–I**). As illustrated, treatment of these cells with SCZ significantly decreased their confluence rate. The effect of SCZ was more efficient in the presence of low concentration of serum. We next evaluated the effect of SCZ on the migration of these cancer cells in a wound-healing assay. As illustrated in **Figures 6A–D**, treatment with SCZ for indicated time periods, reduced cancer cell motility in a concentration and time-dependent manner.

### 3.6 Sulconazole inhibits tumor growth in zebrafish

**Figure 7:**
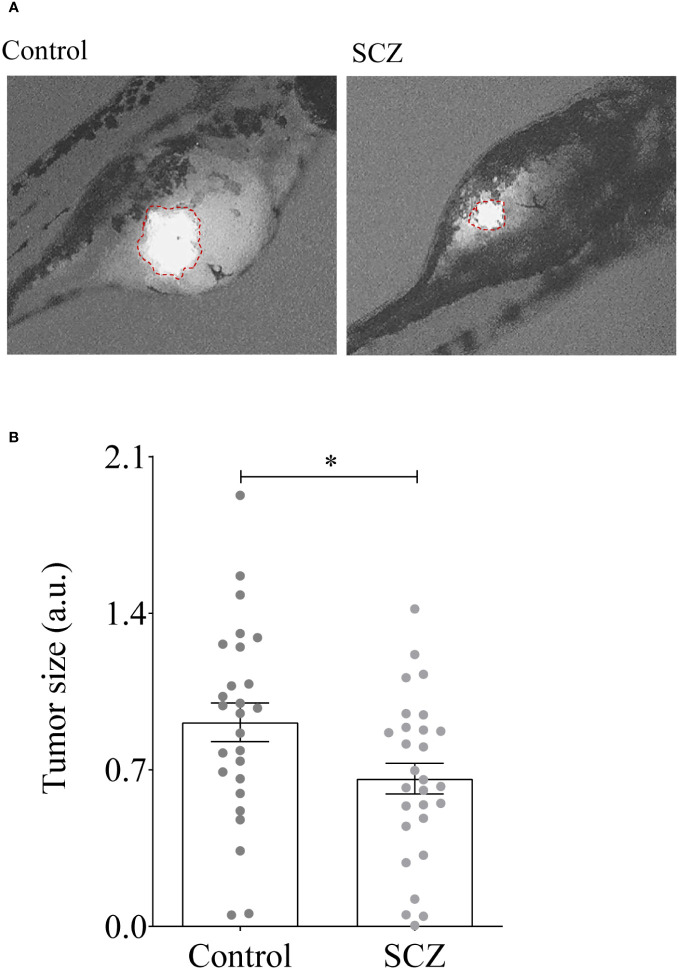
Inhibition of tumor growth by Sulconazole. **(A, B)** MDA-MB-231 cells (500) were injected above the duct of Cuvier in perivitelline space of the embryos and SCZ (1 µM) or vehicle (DMSO) were added to embryo E3 medium. Tumor imaging was performed after 48-h post injection. Results are representative of 4 experiments. Values are mean ± SEM (*n =* 25-30 per group). *P < 0.05.

To directly assess the effect of SCZ on tumor growth, we used zebrafish embryos injected with MDA-MB-231 cancer cells, as *in vivo* model. Indeed, the adaptive immune system of zebrafish matures at 28 dpf (25–27). Previously, the development of T cells in zebrafish was reported to occur post 5 days post-fertilization (dpf). This temporal sequence corresponds to the infiltration of the thymus by lymphoid progenitor cells around c with subsequent egress from the thymus occurring between 6 dpf and 7 dpf, facilitating entry into the peripheral circulation (28). Thereby, multiple cancer models have been generated in zebrafish and proven similar to their human counterparts molecularly and pathologically (22, 29, 30). To assess the effect of SCZ on tumor growth we used 2 dpf zebrafish embryos to inject 500 cells/fish (25-30 fish) and allowed to grow for 48h in the absence and presence of SCZ (1μM). As shown in **Figures 7A, B**, the presence of SCZ in the embryo’s media reduced up to ~ 4-fold time MDA-MB-231 cancer cells ability to induce tumor growth in zebrafish embryos, confirming the antitumorigenic effect of SCZ *in vivo*.

## 4 Discussion

The interaction between PD-1 on T cells and its ligand PDL-1 expressed on tumor cells directly affects the cytotoxic function of T cells required for cancer cells eradication (1, 6). However, the tumor microenvironment (TME) including the components of the extracellular matrix of various TME cells affect the expression of PD-1 on T cells leading to immune evasion. The monoclonal antibodies used to target PD-1 and PD-L1 were found to be potent immune checkpoint inhibitors and were used for various cancers treatment such as melanoma, lung cancer and gastric cancer. Indeed, since 2014, FDA has approved various anti-PD-1 and anti-PD-L1 monoclonal antibody drugs. These drugs have made serious improvement in the clinical treatment of various tumors and prolonged the survival of cancer patients. For several cancer patients, these drugs mediated complete remission. However, other patients with solid tumors, including colorectal cancers [except the microsatellite-instable (MSI) subset] are refractory to these treatments (6, 8).

The failure to respond to anti-PD-1 drug is mainly due to the presence of irreversibly exhausted T cells. In addition, various adverse responses are mediated by these inhibitors that are mainly immune-related adverse reactions found to affect several tissues and organs of the treated patients (31, 32). Other studies also showed that it is difficult for antibody drugs to successfully infiltrate tumor tissues and reach all areas of the tumor microenvironment in order to be accumulated at an adequate and efficient concentration (17). Other studies revealed that the efficacy of these drugs can be reduced with time and was linked to their immunogenicity that induces the production of anti-antibodies during the treatment (32). Therefore, it is necessary to develop novel inhibitors such as small molecules with confirmed efficacy and safety to target the PD-1/PD-L1 interaction. Indeed, compared to monoclonal antibodies that are difficult to produce, small molecule present various advantages to make them promising for clinical treatments (33). Small molecule inhibitors are more appropriate for oral management, and can be used to avoid immune-related adverse effects due to the facility of their changeable half-life. In this study, we demonstrate that SCZ is able to represses the expression of PD-1 on T cells and PBMCs and reduces cancer proliferation, migration and tumor growth *in vivo* using zebrafish embryos model.

Previously, NF-κB and calcium signaling pathways were reported to be required for PD-1 expression on T cells (17). We found that SCZ inhibited these pathways and thereby linking PD-1 repression by SCZ in T cells to NF-κB activity and calcium mobilization inhibition. Further analysis revealed that SCZ is also able to repress CTLA-4 expression in PBMCs. Our results identified SCZ, as small not toxic molecule medicine that may constitute a starting point for the identification of new class of immune checkpoint inhibitor regulators that will have the chance to be used at the clinical setting since their none toxicity was previously established. Indeed, current preclinical studies revealed that small molecule compounds have a better capability than antibodies to repress tumor growth progression (25), suggesting their use to overcome the existing problems of antibody drugs and allow them to replace monoclonal antibodies or serve as complementary therapies.

## 5 Conclusions

Innovative pharmacological methods are needed to improve treatments of advanced cancers, regardless of the considerable benefit of immunotherapy. Indeed, new drugs are needed for to complement the used monoclonal antibodies to target PD-1 and/or PD-L1 immune checkpoint proteins. Here, we have identified SCZ a known antifungal drug which could reinforce immunotherapy based on their capacity to modulate PD-1 expression. We revealed that SCZ repress PD-1 expression on activated T cells through NFκB and calcium mobilization inactivation. It will be of additional interest to explore whether SCZ induces T cells infiltration in tumors and mediates synergic effect with the current immunotherapy.

## Data availability statement

The raw data supporting the conclusions of this article will be made available by the authors, without undue reservation.

## Ethics statement

The studies involving humans were approved by Bergonié Institute (Bordeaux, France). The studies were conducted in accordance with the local legislation and institutional requirements. The human samples used in this study were acquired from a by- product of routine care or industry. Written informed consent for participation was not required from the participants or the participants’ legal guardians/next of kin in accordance with the national legislation and institutional requirements.

## Author contributions

SP: Conceptualization, Methodology, Writing – original draft. MT: Conceptualization, Investigation, Methodology, Writing – review & editing. IG: Conceptualization, Methodology, Writing – original draft. SE: Conceptualization, Methodology, Writing – original draft. IB: Formal analysis, Writing – review & editing. FD: Investigation, Methodology, Writing – review & editing. DF: Writing – review & editing. TS: Investigation, Methodology, Writing – original draft, Writing – review & editing. GS: Investigation, Methodology, Writing – original draft, Writing – review & editing. BV: Conceptualization, Writing – original draft, Writing – review & editing. A-MK: Conceptualization, Funding acquisition, Methodology, Supervision, Writing – original draft, Writing – review & editing.
