# Supplementary figures and images for: Sulconazole inhibits PD-1 expression in immune cells and cancer cells malignant phenotype through NF-κB and calcium activity repression
Source: Front Immunol. 2024 Jan 5;14:1278630. doi: 10.3389/fimmu.2023.1278630 (PMC10796450; doi:10.3389/fimmu.2023.1278630)

## MDA-MB 231

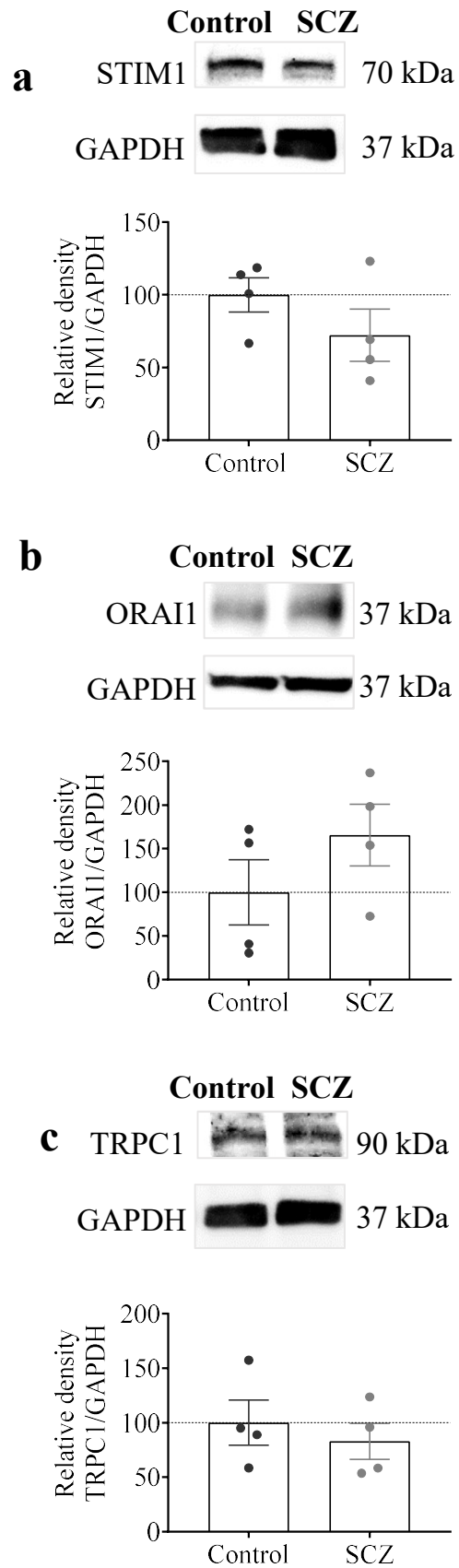

Supplementary Fig. 1

Supplement: Supplementary file 1 [file DataSheet_1.pdf]
